# Estimating Organism Abundance Using Within‐Sample Haplotype Frequencies of eDNA Data

**DOI:** 10.1111/1755-0998.70104

**Published:** 2026-02-13

**Authors:** Pedro F. P. Brandão‐Dias, Gledis Guri, Megan R. Shaffer, Elizabeth Andruszkiewicz Allan, Ryan P. Kelly

**Affiliations:** ^1^ School of Marine and Environmental Affairs University of Washington Seattle WA USA

**Keywords:** alleles, individuals, likelihood, metabarcoding, population genetics

## Abstract

Environmental DNA (eDNA) provides powerful insights into species presence and community composition but remains limited in its capacity to infer species abundance or population structure. Here, we show that the deviation between within‐sample haplotype frequencies and the overall population‐level haplotype frequencies can be used to estimate the number of individual contributors to a given sample. We first establish the theoretical framework for approximating population haplotype frequencies directly from eDNA data, enabling application even in the absence of tissue‐derived references. Building on this foundation, we introduce a maximum likelihood estimator to infer the number of contributors and assess its performance through simulations spanning a range of haplotype frequency distributions and noise scenarios. These approaches assume that all samples are drawn from a single, panmictic population. We find that accurate estimates are attainable when haplotypes are sufficiently variable, population frequencies are well‐characterised, and samples are large enough to capture frequency deviations. By bridging population genetic theory and eDNA, our method complements existing molecular approaches and offers a novel path towards quantifying abundance from eDNA metabarcoding data.

## 1 Introduction

Most vertebrate environmental DNA (eDNA) studies have generally focused on either single‐species PCR, which enables precise detection and quantification of target taxa eDNA, or on metabarcoding, which provides broader snapshots of community composition but typically yields only proportion data (Bylemans et al. 2019; McColl‐Gausden et al. 2023; Pont et al. 2023). Metabarcoding can be applied either to bulk samples (e.g., homogenised tissues, gut contents) or to eDNA from environmental samples (e.g., water, soil, air), which differ in ecology and interpretation (Macher et al. 2018) – here, we focus on environmental samples. Although both approaches have greatly advanced our understanding of biodiversity, the limited quantitative information available from eDNA data hampers our ability to accurately monitor species abundances and dynamics at larger scales (Guri, Shelton, et al. 2024; Pont et al. 2023). Thus, obtaining more robust quantitative information from eDNA data could enable the ability to detect and monitor broad sections of biodiversity simultaneously.

While read counts in metabarcoding data are occasionally interpreted quantitatively in the literature, and superficial correlation between sequencing read number and species abundance may exist (Elbrecht and Leese 2015; Lamb et al. 2022), even under identical sequencing depths, the number of reads assigned to a particular organism is affected by multiple sources of observation error (Gold et al. 2023; Shaffer et al. 2025), which may lead to incorrect interpretations. These errors can be separated into those affecting how well sequencing reads reflect the quantity of DNA molecules present in a sample (observation error) and those affecting the relationship between DNA quantity and organism abundance (biological variability). A key source of observation error is amplification bias, which is due to how well a given organism's DNA is amplified in PCR, often as a result of sequence mismatches with primers (Shaffer et al. 2025; Sipos et al. 2007). This can significantly alter the representation of species proportions in metabarcoding results (Elbrecht and Leese 2015; Polz and Cavanaugh 1998; Shelton et al. 2023). Additionally, because metabarcoding data are compositional, an increase in the amount of DNA of one organism within the sample will reduce the read depth and proportion of reads for other organisms, even if their absolute DNA quantities remain unchanged (Elbrecht and Leese 2015; Guri, Shelton, et al. 2024; Shelton et al. 2023).

Recent statistical advances have allowed for more accurate and simultaneous assessments of DNA quantities of multiple organisms from metabarcoding data (Allan et al. 2023; Guri, Shelton, et al. 2024; Shelton et al. 2023). However, even when DNA quantities are estimated precisely, biological variability that changes the link between total eDNA and organism abundance remains. This link is a complex function of biomass, metabolism, system‐specific biotic and abiotic factors and the spatiotemporal dynamics of eDNA decay and transport (Yates et al. 2025). Between‐species comparisons add further complication because of inherently different shedding rates across taxa (Andruszkiewicz Allan et al. 2021; Rourke et al. 2022). Therefore, obtaining absolute quantities of DNA within environmental samples, be that by quantitative PCR or correcting metabarcoding data, is still several steps away from determining organism abundance or biomass in a region. Thus, although semi‐quantitative analyses of metabarcoding data often provide valuable insights (Guri, Westgaard, et al. 2024), metabarcoding read counts should not be used for quantitative analyses without prior corrections and especially not interpreted as organism abundance (Elbrecht and Leese 2015; Kelly et al. 2019; Shelton et al. 2023).

However, metabarcoding data offer more information beyond species composition and read counts. They also contain within‐species genetic variation, because a single environmental sample can include multiple haplotypes (or amplicon sequence variants, ASVs) from different individuals of the same species, a signal that carries relevant information but is not often explored (Serrana and Watanabe 2023; Tsuji et al. 2020). How much DNA each individual organism contributes to an eDNA sample will depend on several factors including their eDNA shedding rate (Maruyama et al. 2014; Yates et al. 2021) and the distance in space and time between target organism and sampling location (Harrison et al. 2019; Laporte et al. 2020). Nonetheless, when individuals possess different alleles at a given locus, the eDNA sample will contain a mixture of these alleles (Shi et al. 2025), with their expected frequencies proportional to the contributions of each individual.

**FIGURE 1:**
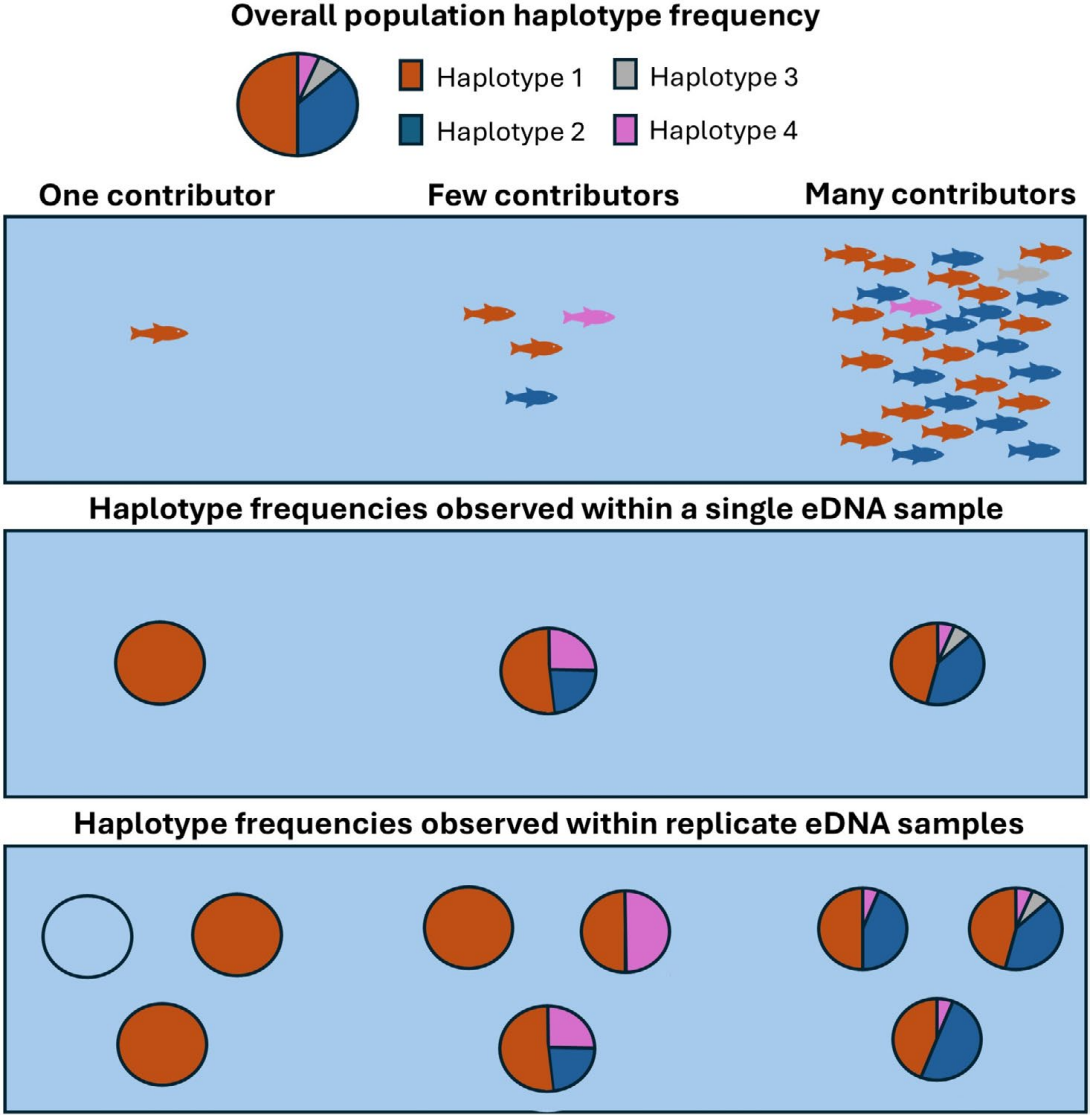
Convergence of observed eDNA haplotype frequencies towards population frequencies with increased number of contributors. The pie chart on the top left depicts the population haplotype frequencies, corresponding to the distribution of haplotypes (haplotypes 1–4) represented by colours. With fewer contributors, the observed haplotype frequencies are quite different from the population frequencies, but with more contributors, eDNA from a greater diversity of individuals is captured and observed haplotype frequencies increasingly align with population frequencies (see also Figure S1). Thus, with a single eDNA sample, the difference between within‐sample haplotype frequencies and the population frequencies can be used to infer the number of contributors. When multiple samples are available, the difference between samples will also offer an additional level of information regarding the variability of the observation process, and estimations can be made more precisely. The leftmost empty circle in the bottom panel represents no detection at one of the replicates.

Under panmixia, meaning random mating within an unstructured population, the haplotypes detected in an eDNA sample would represent a multinomial draw from the population's haplotype frequency distribution (Kingman 1982; Wright 1931), much like drawing coloured marbles from a jar. As the number of individuals contributing eDNA to a sample (hereafter referred to as ‘contributors’) increases, more alleles are likely to be detected (Ai et al. 2025; Wakimura et al. 2023), and the allele frequencies observed within the sample are expected to converge towards the population frequencies of those alleles, as illustrated in Figures 1 and S1. We therefore hypothesise that the difference between haplotype frequencies measured in eDNA samples and those expected from the population reflects the number of contributors, with larger differences indicating fewer contributors and smaller differences indicating many.

Similar but distinct principles have been previously applied to eDNA samples (Ai et al. 2025; Andres et al. 2021; Yoshitake et al. 2019). The core realisation behind all of these methods is that an eDNA sample is a complex, mixed genetic signal from multiple contributors, but these methods rely on different subsets of the information encoded in the haplotypes detected. Conceptually, a mixed eDNA sample provides three tiers of information: (1) the number of distinct haplotypes present, (2) the identity of those haplotypes and (3) their observed frequencies within the sample. The first tier relies only on haplotype count – how many distinct haplotypes are detected without considering their identities or frequencies. With this, Yoshitake et al. (2019) demonstrated that the number of haplotypes can be used to infer the number of individuals in an eDNA sample. More recently, Ai et al. (2025) further demonstrated that the number of haplotypes correlates with the number of individual contributors, making a frequency‐agnostic estimator of contributor abundance. While this method is elegant in its simplicity, its informativeness depends on the underlying (but unknown) haplotype frequency distribution, and it cannot generalise well across markers or populations with varying allele frequencies.

The second tier of information, the identity of haplotypes, was previously explored to determine the number of contributors to eDNA samples by Andres et al. (Andres et al. 2021; Andres, Lodge, and Andrés 2023; Andres, Lodge, Sethi, and Andrés 2023). Their method builds on earlier work with mixed genetic samples from forensic settings to determine the likelihood of mixture given population frequencies of alleles (Haned et al. 2011; Sethi et al. 2019; Weir et al. 1997). The method does not consider the frequency of haplotypes within samples but only their identity and respective known population frequencies. Therefore, it assumes that within‐sample frequency information is irrelevant – a fair assumption in a forensic scenario where the method originates (Paoletti et al. 2005; Weir et al. 1997). While this model may be particularly useful when frequency data are extremely noisy, such as under strong stochastic effects or technical error, if allele frequencies are too noisy to be informative, then treating presence/absence as more reliable is equally problematic, especially for rare alleles. Thus, it would be desirable to use frequency information while still considering haplotype identity. A key methodological advance introduced here is the use of the third tier of information in the within‐sample haplotype frequencies.

A fundamental requirement of methods exploring the second and third tiers of information is knowledge of the population's haplotype or allele frequencies (*π*), a level of detail rarely available for organisms detected through eDNA metabarcoding. In practice, this creates a conceptual paradox: if a population has been extensively sampled to estimate haplotype frequencies, then its abundance is likely already known, making further eDNA‐based inference redundant. However, numerous independent studies over the years have demonstrated that population frequencies can be potentially inferred directly from eDNA data. For example, Sigsgaard et al. (2016) showed that haplotypes recovered from multiple whale shark eDNA detections matched frequencies observed in tissue samples. Uchii et al. (2016) found that carp haplotype frequencies from environmental samples aligned with those in controlled systems. Andres, Lodge, and Andrés (2023) also recovered accurate nuclear and mitochondrial alleles for gobies, though eDNA also sampled rare haplotypes that were absent in tissue frequencies. Wakimura et al. (2023) showed that haplotype frequencies of an endangered fish species recovered from eDNA matched those of traditional sampling after proper filtering. In the subsequent study, Wakimura et al. (2025) found again that eDNA‐recovered mtDNA haplotypes of an invasive fish matched the tissue‐derived frequencies from previous studies. Finally, Parsons et al. (2025) found no bias in the frequency of haplotypes of porpoises generated from eDNA samples when compared to tissue samples.

Beyond eDNA's repeated success in recovering population haplotypes in empirical studies where tissue‐derived frequencies were available to verify its effectiveness, there are strong theoretical reasons to expect that eDNA samples capture a representative snapshot of a population's genetic diversity. As discussed above, eDNA in any environmental sample is inherently a mixture of DNA from multiple individual contributors. When samples are taken deliberately near a particular individual, such as in Parsons et al. (2025) or in Dugal et al. (2022), who sample fluke prints of large marine animals, the haplotypes recovered may reflect that nearby individual's genetic material, meaning the presence of haplotypes is informative, but their frequencies are not. However, when samples are evenly dispersed across space, capturing DNA from many individuals, these spatiotemporal biases are averaged out, and both the presence and relative frequencies of haplotypes can provide meaningful information. In such cases, each sample reflects an indirect, uneven, and stochastic draw from the population's genetic pool, shaped by local density, movement and shedding dynamics. Hence, the average frequency of haplotypes across multiple eDNA samples should be a fair approximation of the population frequencies, provided enough samples are drawn and that there is no haplotype‐specific observation bias.

In light of these theoretical expectations, we present an approach to estimate the population frequency of haplotypes with eDNA samples, and subsequently estimate the number of contributors to each eDNA sample. These methods rely exclusively on the observed within‐sample frequencies, expanding the utility of metabarcoding data beyond presence‐absence assessments to provide estimates of organismal abundance in mixed samples when multiple ASVs are identified for a target organism. In this study, we describe the methods in detail and validate their potential and limitations using simulated eDNA data. While not a direct measure of absolute abundance, this framework complements other quantitative methods by offering additional insights into the interpretation of eDNA metabarcoding results.

## 2 Methods

As described above, the variability of haplotype frequencies within eDNA observations relative to population frequencies carries information about the number of contributors (*N*) to each sample. We extract this information in two steps. First, we use the full, spatially replicated eDNA dataset to derive a population‐level haplotype frequency vector *π*: each sample contributes equally to a leave‐one‐out mean (see below). Second, we offer a normal approximation maximum‐likelihood estimator (MLE) to obtain both a point estimate and confidence interval for *N*. The sections below will walk through these derivations.

### 2.1 Common Mathematical Framework

**FIGURE 2:**
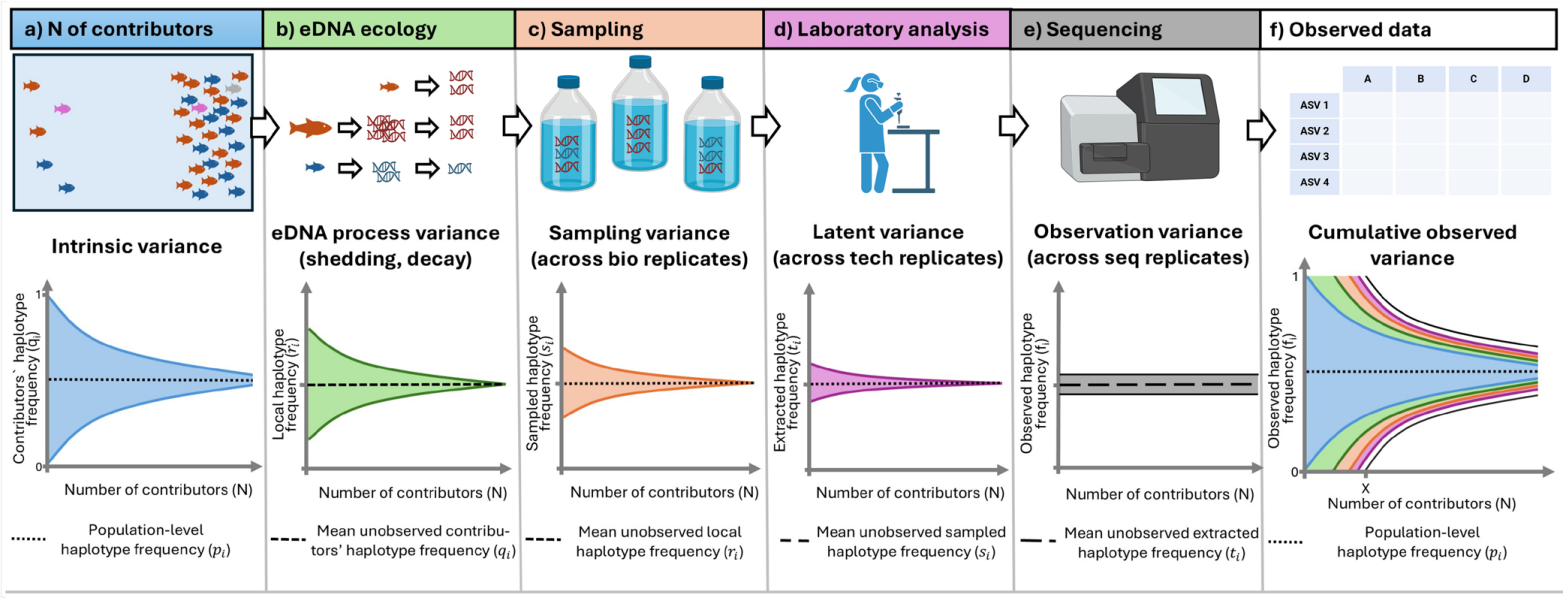
Propagation of variance in haplotype frequencies observed within an environmental DNA sample. Panel (a) illustrates the inherent process of eDNA production and distribution in the natural environment, where the variability in haplotype frequencies is intrinsically higher in regions with fewer contributors (*N*). This baseline variance allows in theory to infer the original number of contributors by examining haplotype frequency variability. In panel (b) we observe the eDNA process variance, where different individuals (and thus biomasses) shed different quantities of DNA, or distance (in space or time) between an individual contributor and the sample results in reduced eDNA concentrations from either decay or transport. Thus, individual contributors provide a variable amount of eDNA to the local eDNA pool, affecting local haplotype frequencies (*r*_*i*_). By ‘local eDNA pool’, we mean the entire eDNA pool in a given area that could be sampled. Panel (c), eDNA is sampled, yielding intermediate, unobserved sampled frequencies for each haplotype (*s*_*i*_). Panel (d) depicts the laboratory processes such as DNA extraction and pipetting that further add noise, resulting in extracted haplotype frequencies (*t*_*i*_). In panel (e), the sequencing step is represented resulting in observed haplotype frequencies (*f*_*i*_). Finally, panel (f) shows that the final observed haplotype frequencies incorporate the cumulative variance from all stages – from natural eDNA distribution through sampling, laboratory processing and sequencing. Point X on the x‐axis shows the N that is indistinguishable from 1 due to added noise. A more detailed mathematical breakdown of each process is available in Figure S2 and Table S1. Created with BioRender.

Let *π =* (*p*_*i*_,*…, p*_*K*_) denote the population frequencies of haplotypes $i\in \left\{1,\ldots k\right\}$ at the same locus, such that all the frequencies sum to 1. We assume that independent eDNA samples *j* have been collected and denote by $N_{j}$ the total (unknown) number of contributors to sample *j*. Under panmixia, the haplotypes contributed to each sample can be viewed as a multinomial draw:

(1) $$n_{\mathrm{ij}}\sim \text{Multinomial}\;\left(N_{j},\pi \;\right)$$

where $n_{\mathrm{ij}}$ is the unobserved number of contributors in sample *j* that carry haplotype *i*. Thus, the expected frequency of haplotype *i* among contributors to sample *j* is

(2) $$\begin{array}{c} q_{\mathrm{ij}}=\frac{n_{\mathrm{ij}}}{N_{j}},\text{with}\;\mathrm{Var}\left(q_{\mathrm{ij}}\right)=\frac{p_{i}\left(1-p_{i}\right)}{N_{j}} \end{array}$$

where $p_{i}$ is the population frequency of haplotype *i*. The haplotype frequency $q_{\mathrm{ij}}$ contains the signal of interest for estimating $N_{j}$ through its variance. However, $q_{\mathrm{ij}}$ is unobservable from eDNA samples. Instead, we observe a downstream quantity: the number of sequencing reads for each haplotype, $R_{\mathrm{ij}}$, and the frequency of each, $f_{\mathrm{ij}}$. It is important to understand that these observed frequencies are not the same as $q_{\mathrm{ij}}$; they are influenced by a sequence of ecological and technical processes that introduce additional noise to the original haplotype frequencies present among the contributors (Figure 2).

Altogether, the variance in the final observed frequencies *f*_*ij*_ reflects the cumulative effect of all downstream processes following individual DNA shedding (Figure 2f). As illustrated in Figures 2 and S2, successive ecological (Barnes and Turner 2016; Yates et al. 2025), sampling, laboratory and sequencing processes add stochastic variance to haplotype frequencies, inflating the variance beyond the baseline contributor‐level expectation. Critically, these sources of noise are not necessarily biased with respect to the haplotype frequencies or identities present in the contributing individuals. This is the key assertion, and if it holds, then:

(3) $$\begin{array}{c} E\;\left[f_{\mathrm{ij}}\right]=q_{\mathrm{ij}} \end{array}$$

That is, the expected observed frequency still reflects the true underlying frequency among contributors – a quantity not otherwise observable – even if the observed values deviate due to added noise. In this view, the observed frequencies $f_{\mathrm{ij}}$ are conditionally unbiased estimates of the unobserved $q_{\mathrm{ij}}$, but with added variance.

To make this variance explicit on the proportion scale, we combine all added variance into a single multiplicative inflation factor ξ≥1 that acts on the $N_{j}$ term, such that as the number of contributors decreases, we observe proportionately more variance in the allele frequencies:

(4) $$\begin{array}{c} \mathrm{Var}\left(f_{\mathrm{ij}}\right)=\xi \frac{p_{i}\left(1-p_{i}\right)}{N_{j}} \end{array}$$

where $p_{i}$ is the ‘true’ population frequency of haplotype *i* and $N_{j}$ is the number of contributors to sample *j*. Intuitively, ξ=1 retains only contributor haplotype sampling variance (Figure 2a), while ξ>1 inflates that variance to account for downstream variance (Figure 2b–e). The key hypothesis is that the $\frac{1}{N_{j}}$ term remains the dominant component, such that the original number of contributors remains inferable. In practice, this requires that the number of DNA molecules captured and the sequencing depth is sufficient to preserve haplotype frequency information. If low recovery steps occur – for example, due to small sampling volumes, poor extraction efficiency, or shallow sequencing – then downstream stochasticity dominates, haplotype frequencies become effectively random and inference becomes meaningless. Biological replicates can help diagnose and mitigate this issue: if replicate samples from the same site yield highly inconsistent haplotype frequencies, it is likely that technical noise is overwhelming the contributor signal.

### 2.2 Assumptions and Requirements

Beyond the requirement for known allele frequencies, and that observation noise is not overwhelming, the method depends on other key assumptions to be laid out explicitly. First, it requires panmixia (i.e., no population structure within the study area) and that loci used are in Hardy–Weinberg Equilibrium. These assumptions are crucial because processes such as local adaptation or population structure could cause certain alleles to be overrepresented in certain samples for reasons unrelated to sampling. Second, it is assumed that ASVs observed in eDNA data correspond to real haplotypes in the population and are not false alleles (e.g., due to amplification or sequencing errors). These are potential concerns, as they have been observed in eDNA data (Elbrecht et al. 2018; Macé et al. 2022; Tsuji et al. 2020). Numerous approaches to correct for such errors are available and should be applied to data before interpreting observed ASVs as haplotypes (Frøslev et al. 2017; Jeunen et al. 2024; Koseki et al. 2025).

### 2.3 Deriving Population Haplotype Frequencies

Under the multinomial model described above (Equation 1), the observed frequency of haplotype *i* in sample *j* $\left(f_{\mathrm{ij}}\right)$ is a random variable with $E\;\left[f_{\mathrm{ij}}\right]=p_{i}$. Thus, because each observed $f_{\mathrm{ij}}$ is an unbiased draw centred on $p_{i}$, averaging across multiple independent samples provides a natural estimator of the true population frequency:

(5) $$\begin{array}{c} \hat{p_{i}}=\frac{1}{M}\sum_{j=1}^{M}f_{\mathrm{ij}\;} \end{array}$$

where M is the total number of samples where any target ASV is detected. This estimator is unbiased, because $E\;\left[\hat{p_{i}}\right]=p_{i}$. Thus, when multiple samples are considered, ${\hat{p}}_{i}$ approximates $p_{i}$. However, importantly, this averaging approach implicitly assumes that all samples contribute equally to the estimate of *π* – that is, it assumes approximate equivalence of N_j_ across samples. Although weighting by sequencing depth is tempting, read counts are compositional and confounded by total DNA, normalisation and run effects; we therefore use an equal‐weight average as a conservative default.

A practical concern arises when the same sample is used to both estimate *π* and infer its own number of contributors using the methods described below, creating circularity. To avoid this, we suggest a leave‐one‐out (LOO) approach: when estimating the number of contributors for a given sample *j*, we compute *π* ^(−j)^ (Equation 5) the estimate of population frequencies derived from all samples except *j*. This ensures that the reference population frequencies used in estimating contributors to sample *j* are independent of the observed frequencies in that same sample.

### 2.4 Estimating Number of Contributors to Individual Samples

Once population haplotype frequencies are known, we may derive the number of individual contributors given the variability in observed frequencies. A statistically rigorous way to model the number of contributors in mixed eDNA samples would be to use a hierarchical model that explicitly accounts for variance added at each sampling stage highlighted in Figure 2, and in further detail in Figure S2. But in practice, the high level of replication required for a hierarchical approach is rare in most datasets, limiting the model's identifiability and applicability. However, sampling, latent and observation variances are most likely dependent on the number of molecules in the sample, and not the true number of contributors (N), acting as noise that increases variance but does not erase the relationship between estimated and true number of contributors. This allows us to simplify the model by ignoring these intermediate unobserved stages and their associated variances to directly link observed haplotype frequency to biological variance in contributor numbers in the real world.

### 2.5 Normal‐Approximation Log‐Likelihood Approach

Given Equations (1) and (4), by the Central Limit Theorem (CLT), each observed frequency $f_{\mathrm{ij}}$ can be approximated by a normal distribution with mean based on the population frequency $p_{i}$ and variance given by the multinomial process (Iliadis et al. 2012; Zhang et al. 2008):

(6) $$\begin{array}{c} f_{\mathrm{ij}}∼\text{Normal}\;\left(p_{i},\xi \frac{p_{i}\left(1-p_{i}\right)}{N_{j}}\right) \end{array}$$

Under this assumption, then the probability density function (PDF) given by the normal distribution and the variance term is

(7) $$\begin{array}{c} P\left(f_{\mathrm{ij}}\;,\;N_{j},p_{i}|\xi \right)=\frac{\sqrt{N_{j}/\xi }}{\sqrt{2\pi \;p_{i}\left(1-p_{i}\right)}}\exp\left(-\frac{N_{j}{\left(f_{i}-p_{i}\right)}^{2}}{2\;\xi \;p_{i}\left(1-p_{i}\right)}\right) \end{array}$$

Then, naively assuming independence across k haplotypes, the log‐likelihood is

(8) $$\begin{array}{c} \ln L\left(N_{j}\right)=\frac{k}{2}\ln\left(N_{j}\right)-\frac{k}{2}\ln\left(\xi \right)-\frac{1}{2}\;\sum_{i=1}^{k}\ln\left(2\pi \;p_{i}\left(1-p_{i}\right)\right)-\frac{N_{j}}{2\xi }\;\sum_{i=1}^{k}\frac{{\left(f_{i}-p_{i}\right)}^{2}}{p_{i}\left(1-p_{i}\right)} \end{array}$$

To maximise $\ln L\left(N_{j}\right)$, we differentiate in relation to $N_{j}$ and set to zero. Fortunately, this leads to a closed form solution:

(9) $$\begin{array}{c} N_{j}^{\mathrm{MLE}}=\xi \frac{K}{\sum_{i=1}^{k}\frac{{\left(f_{\mathrm{ij}}-p_{i}\right)}^{2}}{p_{i}\left(1-p_{i}\right)}} \end{array}$$

where $N_{j}^{\mathrm{MLE}}$ is a point estimate for the most likely number of contributors to an eDNA sample *j* given the added variance ξ, total number of haplotypes *K*, a haplotype's frequency within the sample $f_{\mathrm{ij}}$ and the population frequency for that haplotype, $p_{i}$.

Finally, to assess uncertainty in N, we employ a profile likelihood approach (Murphy and Van Der Vaart 2000). Under standard regularity conditions, the likelihood ratio statistic $-2\left[\mathrm{lnL}\left(N\right)-\ln L\left(N_{\mathrm{MLE}}\right)\right]$ is asymptotically *X* ^2^ distributed with 1 degree of freedom. We then define the confidence interval as the set of N values for which

(10) $$\begin{array}{c} \mathrm{lnL}\left(N\right)\geq \mathrm{lnL}\left(N_{\mathrm{MLE}}\right)-\frac{1}{2}\;X_{1,1-\alpha }^{2} \end{array}$$

where $X_{1,1-\alpha }^{2}$ denotes the 100 (1 – α)% quantile of the chi‐square distribution with 1 degree of freedom.

An alternative method to estimate the number of contributors based on a Method of Moments (MoM) approach, rather than a likelihood or normal approximation, is provided in the Supplement (Figure S4). Because it consistently underperformed relative to the normal approximation across most simulated scenarios, we do not present its results in the main text – but it may be useful for other applications.

### 2.6 Ploidy‐Based Minimum Number of Contributors

In addition to the likelihood approach, we also impose a minimum number of contributors to a sample, derived from the ploidy of the locus under analysis similar to the approach used by Shi et al. (2025) and constraints applied by Andres et al. (2021). Specifically, for haploid loci, we require that the estimated number of contributors to an eDNA sample must be at least equal to the number of observed haplotypes. For example, if four haplotypes are observed in the sample, regardless of their relative frequencies, the sample must have been derived from at least four distinct individuals. Similarly, for diploid loci, we define the minimum number of contributors as half the number of observed haplotypes, rounded up. Using the same example, a sample with four observed haplotypes could be explained by as few as two diploid individuals (e.g., two heterozygotes), but never fewer. Finally, in cases where ploidy cannot be inferred – such as with loci affected by heteroplasmy – this constraint can be disabled.

### 2.7 Simulation

To validate our statistical methods, it was necessary to work with samples where the number of contributors was precisely known and controlled. To this end, we developed a series of three R functions (Team, R. D. C 2010) to simulate eDNA data and apply the method as a proof‐of‐concept. These functions allow us to generate synthetic datasets that replicate the key processes influencing eDNA sampling, including individual contributions, decay, allele frequencies and sampling biases. The functions are essential to replicate findings herein and potentially useful for other applications, and they are provided in the supplement and on the associated github repository.

First, the function ‘generate_contributors’ randomly generates a number of individuals contributing to a local eDNA pool. For each individual k, it then assigns: (1) a haplotype identity via a multinomial draw from the user‐defined population frequencies *π*; (2) a body size $S_{k}$ drawn from a user‐specified range; and (3) a distance $d_{k}$ from the sample. Contributors may be generated as groups, in which case contributors share a single distance from the sample, or as ungrouped, in which case each contributor receives an independently sampled $d_{k}$.

Then, the second function, ‘generate_eDNA’, takes in the output of the first function and simulates the process of eDNA generation and sampling from a local pool of contributors while keeping track of individual molecules. The function first calculates the eDNA shed for each contributor based on their body size and an allometric scaling exponent (default β = 0.75; Brown et al. 2004; Jo et al. 2024; Yates et al. 2025). Once the expected shedding rate is determined, the function reduces each contributor's eDNA exponentially based on the distance $d_{k}$ between that individual and the sampling location (Figure S2b). Following these steps, the function places all generated molecules in a ‘local pool’ representing the eDNA shed by potential contributors and that could be collected within a given replicate water sample. This pool keeps track of both the contributor ID and haplotype of each molecule.

The function then simulates the eDNA sampling process by drawing a fraction of the local pool into a ‘bottle replicate,’ representing a subsample of the available eDNA in the local pool. The number of molecules sampled is determined by a user‐defined parameter (‘bottle_volume’), which specifies the proportion of the total local pool that is captured in each replicate. A random subset of molecules is selected without replacement, ensuring a realistic representation of stochastic sampling effects. For each replicate, the function summarises the total number of eDNA molecules captured, the number of unique contributors present and calculates haplotype‐specific relative frequencies.

Finally, a third function, ‘simulate_metabarcoding_data’, takes the output of ‘generate_eDNA’ and simulates high‐throughput sequencing observations (i.e., metabarcoding reads) for each sample. This function draws a specified number of reads per sample using a multinomial distribution, where the probability of each haplotype is proportional to its relative abundance in the simulated eDNA pool. Note that this step does not include subsampling and amplification steps, and is thus a simplification that assumes no amplification bias between haplotypes. Additional stochasticity can be added via an error term applied to the haplotype probabilities before sampling, mimicking noise introduced during laboratory analyses. The function also supports multiple technical replicates per sample.

**FIGURE 3:**
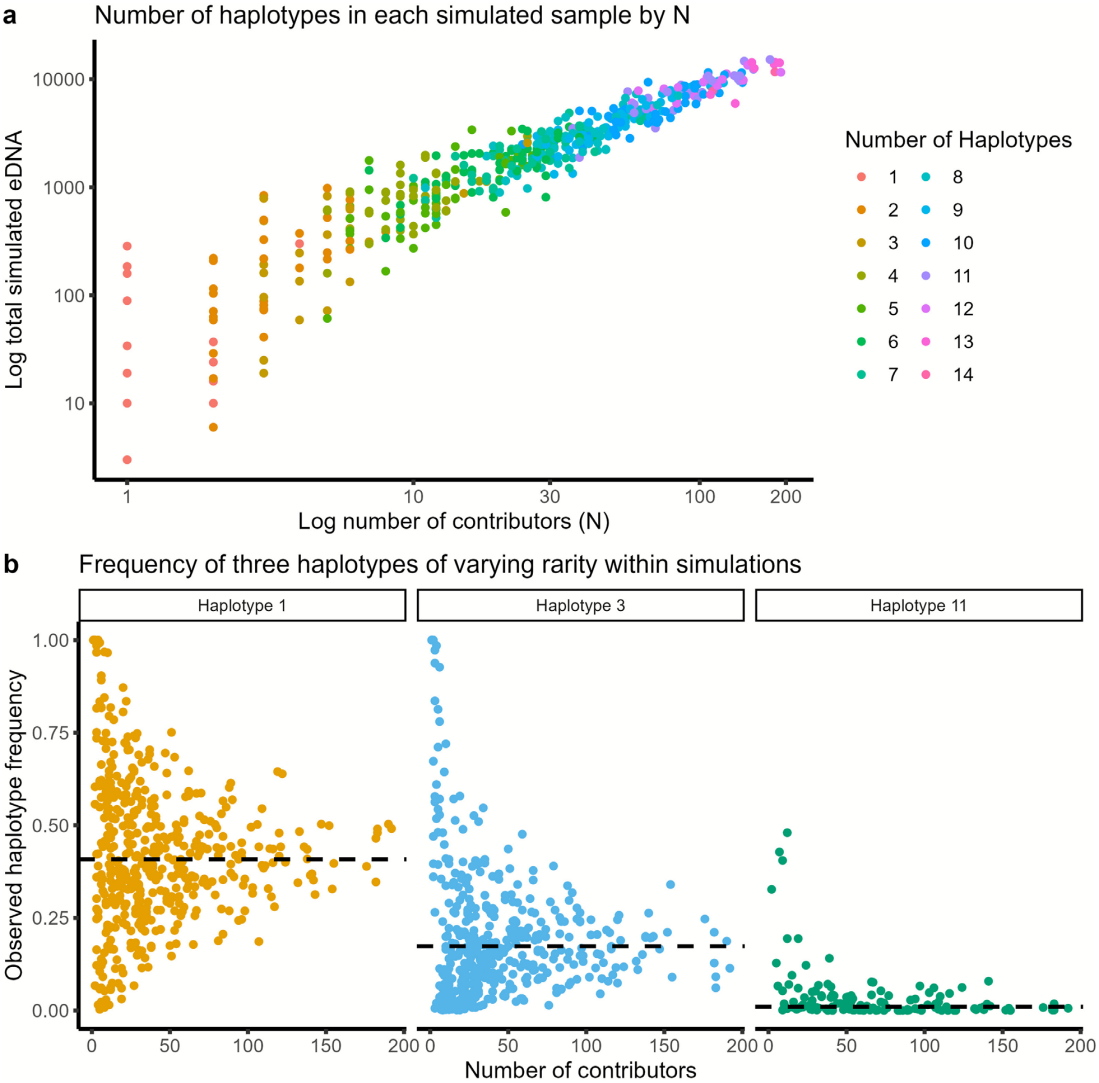
Haplotype patterns recovered from eDNA simulations. Simulations with a hypothetical variable marker with 14 alleles of varying frequency chosen for ease of observation of patterns: *π* = (0.400, 0.170, 0.170, 0.050, 0.050, 0.050, 0.050, 0.010, 0.010, 0.010, 0.010, 0.010, 0.005, 0.005). Here, we have simulated 500 samples with 1–200 contributors to each sample, that vary in size and distance between 1 and 100 (ξ > 1). (a) Total simulated eDNA in each simulated sample by number of contributors with colour showing number of haplotypes observed in each sample. (b) Frequency of three haplotypes of varying frequency within simulations. Dashed black line in each panel is the population frequency set for each haplotype. Haplotype 1 = 0.400; Haplotype 3 = 0.170; Haplotype 11 = 0.010.

**FIGURE 4:**
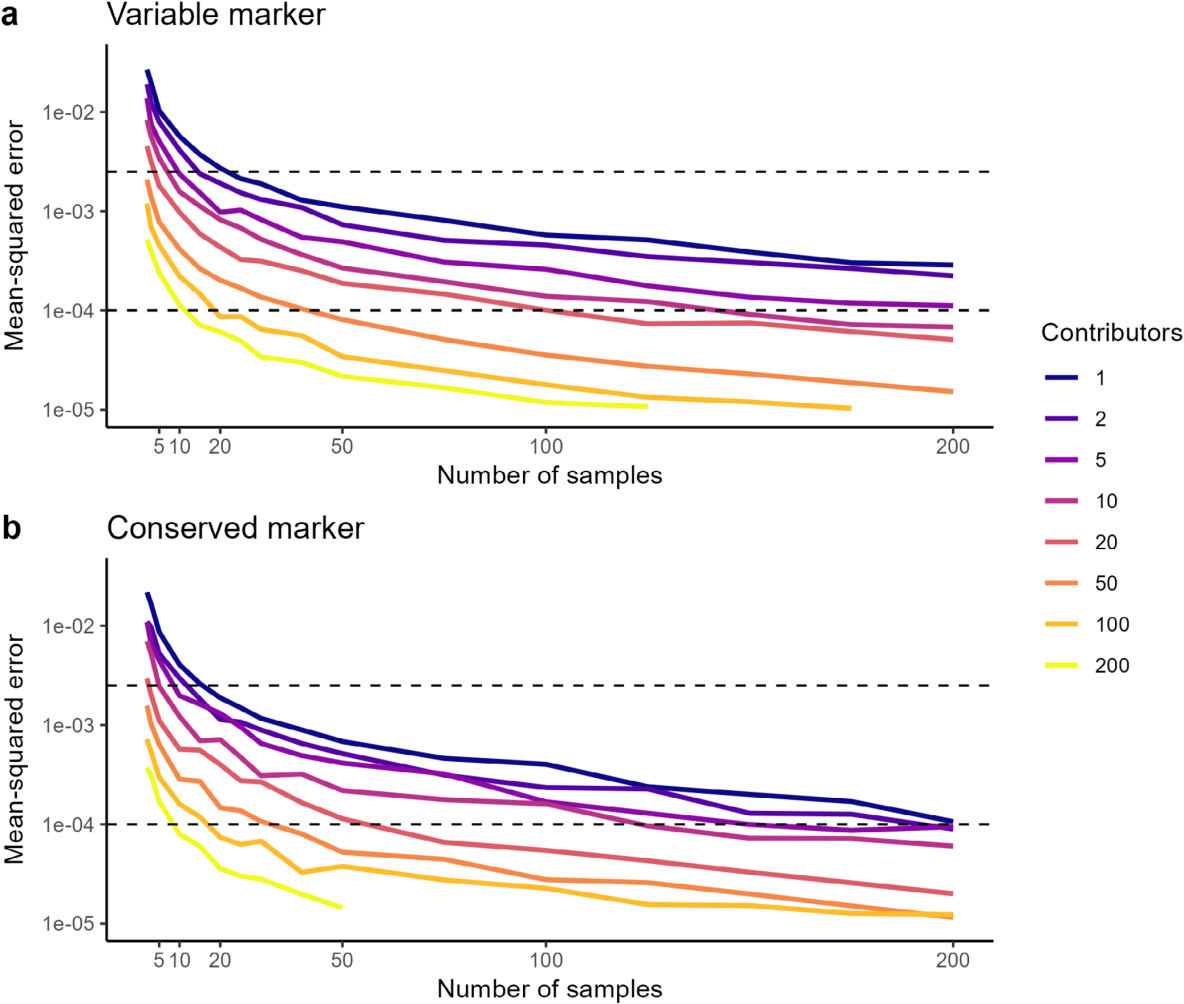
Error of population‐frequency estimates as a function of sampling effort and mean number of DNA contributors. Lines show the mean‐squared error (MSE) between the leave‐one‐out estimates of the population haplotype‐frequency vector, and the true vector (*π*) for an increasing number of independent eDNA samples. For ease of reference, the horizontal lines mark an MSE of 0.0025 (≈5%) and 0.0001 (≈1%). (a) Variable‐frequency mitochondrial marker with 14 haplotypes. (b) Highly conserved mitochondrial marker with 6 haplotypes. In both plots, colours differentiate scenarios with increasing average numbers of individual DNA contributors per sample (legend, right; darker blues = small local populations, lighter yellows = large). Simulations include the same level of variance as those in Figure 3.

**FIGURE 5:**
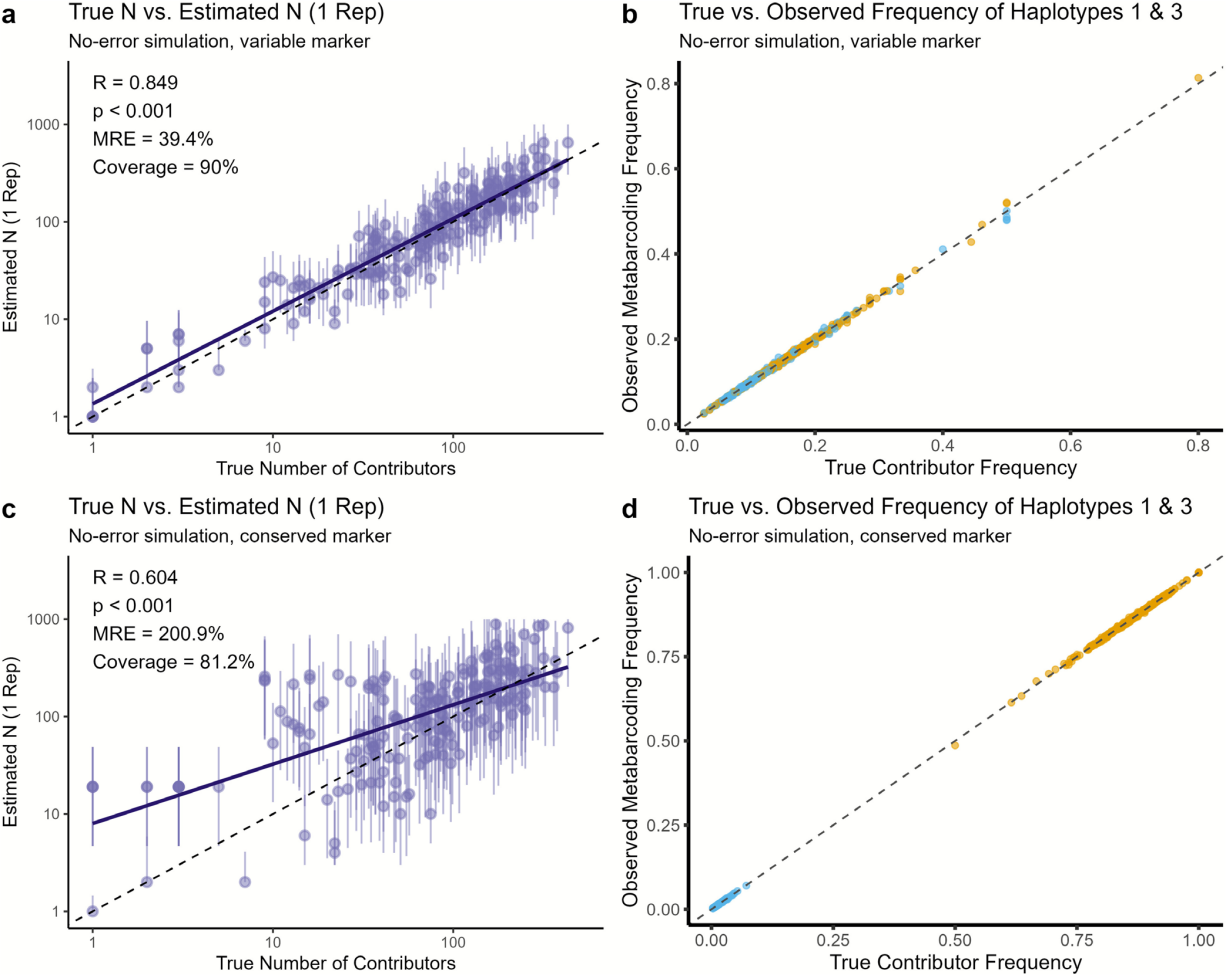
Precision of estimation method applied to simulated data with no added error (ξ = 1). Panels (a, b) show results from a simulation using a hyper‐variable marker with 17 alleles. Panels (c, d) present a simulation using a conserved marker with 6 alleles. Panels (a) and (c) show estimates derived using the Normal Approximation Maximum Likelihood method (Equation 9) compared to the true number of contributors to each simulated sample, where error bars are the estimate 95% CI (Equation 10); panels (b) and (d) show the correlation between simulated contributors haplotype frequencies and the observed haplotype frequencies in the corresponding simulated metabarcoding observations for haplotypes 1 (yellow) and 3 (light blue), demonstrating that in these simulations, little error is added. These haplotypes were selected to represent a variety of frequencies across simulations. Solid lines in all panels represent the linear regression fits, *R* is Pearson's correlation coefficient and dashed lines denote the 1:1 identity. Simulations included 5000 samples. While correlations are devised from all simulations, only a random subset of 200 are plotted here to avoid overplotting.

We conducted two types of simulations in this study: one under idealised ‘no‐error’ conditions (ξ = 1) and another using more biologically realistic parameters (ξ > 1). For the no‐error simulations (Figures 4 and S4), we included an arbitrarily high eDNA shedding rate (rate = 1000), no stochastic error (error = 0), high sequencing depth (100,000 reads), complete sampling of the collection volume (volume = 1), no DNA degradation (decay = 0) and equal DNA contribution from all individuals (no variation in contributor size and distance). For the realistic simulations (Figures 3, 5 and S4), we introduced biologically plausible complexity. These runs used moderate process noise (error = 0.05) and sequencing depth typical for a single species in a community metabarcoding dataset (~1000 reads). We also introduced heterogeneity in individual contributors, with both contributor size and distance to the sampling point drawn from uniform distributions between 1 and 100, and had each bottle replicate sample 10% of the local pool without replacement. Haplotype frequencies used across simulations varied and are specified when relevant below.

## 3 Results

### 3.1 Environmental DNA Data Simulation

Environmental DNA simulations result in a variable number of haplotypes within samples, with more haplotypes observed as the number of contributors increases (Figure 3a). Furthermore, when we look at the variance in observed haplotype frequencies $f_{\mathrm{ij}}$ as a function of the number of contributors (Figure 3b), the pattern depicted in Figure 2 emerges even when individual contributions are heterogeneous (ξ > 1). This difference between observed frequency and population frequency across all haplotypes is, in essence, what the proposed quantitative method uses to derive the most likely number of contributors. However, this trend may not manifest as cleanly in real populations, where genetic structure, redundancy, and environmental processes may obscure haplotype–contributor relationships.

### 3.2 Rapid Estimation of Population Frequencies From eDNA Samples

We assessed how accurately population‐level haplotype frequencies (*π*) can be recovered from simulated eDNA samples alone using the LOO averaging approach. Simulations were performed under two scenarios: a hypothetical hypervariable marker comprising 14 haplotypes *π* = (0.400, 0.170, 0.170, 0.050, 0.050, 0.050, 0.050, 0.010, 0.010, 0.010, 0.010, 0.010, 0.005, 0.005) and a conserved marker dominated by one out of six haplotypes (*π* = 0.86, 0.12, 0.005, 0.005, 0.005, 0.005).

As expected, aggregating haplotype frequencies across spatially replicated eDNA samples reduced stochastic variance, resulting in convergence towards true population frequencies (Figure 4). Increasing the number of simulated samples improved accuracy, and samples with more individual contributors yielded faster convergence (Figure 4). For both marker types, frequency estimates stabilised rapidly: 20 samples were sufficient to recover population frequencies within a mean squared error (MSE) threshold equivalent to 5% deviation across haplotypes, regardless of the number of contributors or haplotype frequencies. When eDNA samples contained more than 10 contributors on average, population frequencies could be estimated within 1% error using as few as 100 samples with target detections. In all cases, eDNA‐derived frequencies approximate true population frequencies more rapidly than tissue samples (Figure 4, one contributor line).

### 3.3 Accurate Prediction of the Number of Contributors in Simulated Data

Once population haplotype frequencies are known, the Normal Approximation Maximum Likelihood approach (Equation 9) can be applied to estimate the number of contributors for each sample. To evaluate the best‐case performance of our approach, we first simulated eDNA data under ideal conditions (Figure 5): all individuals contributed equal amounts of DNA to the local eDNA pool, and no observational error was introduced (ξ = 1, Figure 5b,d). Using haplotype frequencies from a hypothetical hypervariable marker with 17 alleles and *π* = (0.140, 0.120, 0.120, 0.100, 0.100, 0.080, 0.080, 0.06, 0.06, 0.040, 0.040, 0.02, 0.02, 0.005, 0.005, 0.005, 0.005), the method provided accurate estimates that closely matched the true number of contributors (Figure 5a). There was a strong correlation with true values (*R* = 0.849, *p* < 0.001), with 90% of estimates capturing the true value within the 95% confidence interval (coverage; Figure 5a).

We then repeated the simulation using a marker with conserved haplotype characterised by a dominant allele and only 6 total haplotypes *π* = (0.86, 0.120, 0.005, 0.005, 0.005, 0.005). Under these conditions, the method produced strongly biased and less accurate estimates, which tended to be overestimated for lower numbers of contributors (Figure 5c). Correlation with the true number of contributors dropped to *R* = 0.604, mean relative error (MRE) of 200.9% and coverage to 81.2%. This is partially due to overestimation of samples containing only the most common haplotype. Because they have different numbers of contributors but only contain one and the same haplotype, they are all given identical, uniform estimations, which shifts the curve.

These results highlight that even under ideal conditions, the accuracy of contributor estimation is fundamentally tied to haplotype diversity at the locus. As haplotype distributions become increasingly skewed, particularly when one allele dominates, estimation error rises substantially (Figure S3).

### 3.4 Additional Variance Effects on Estimations

**FIGURE 6:**
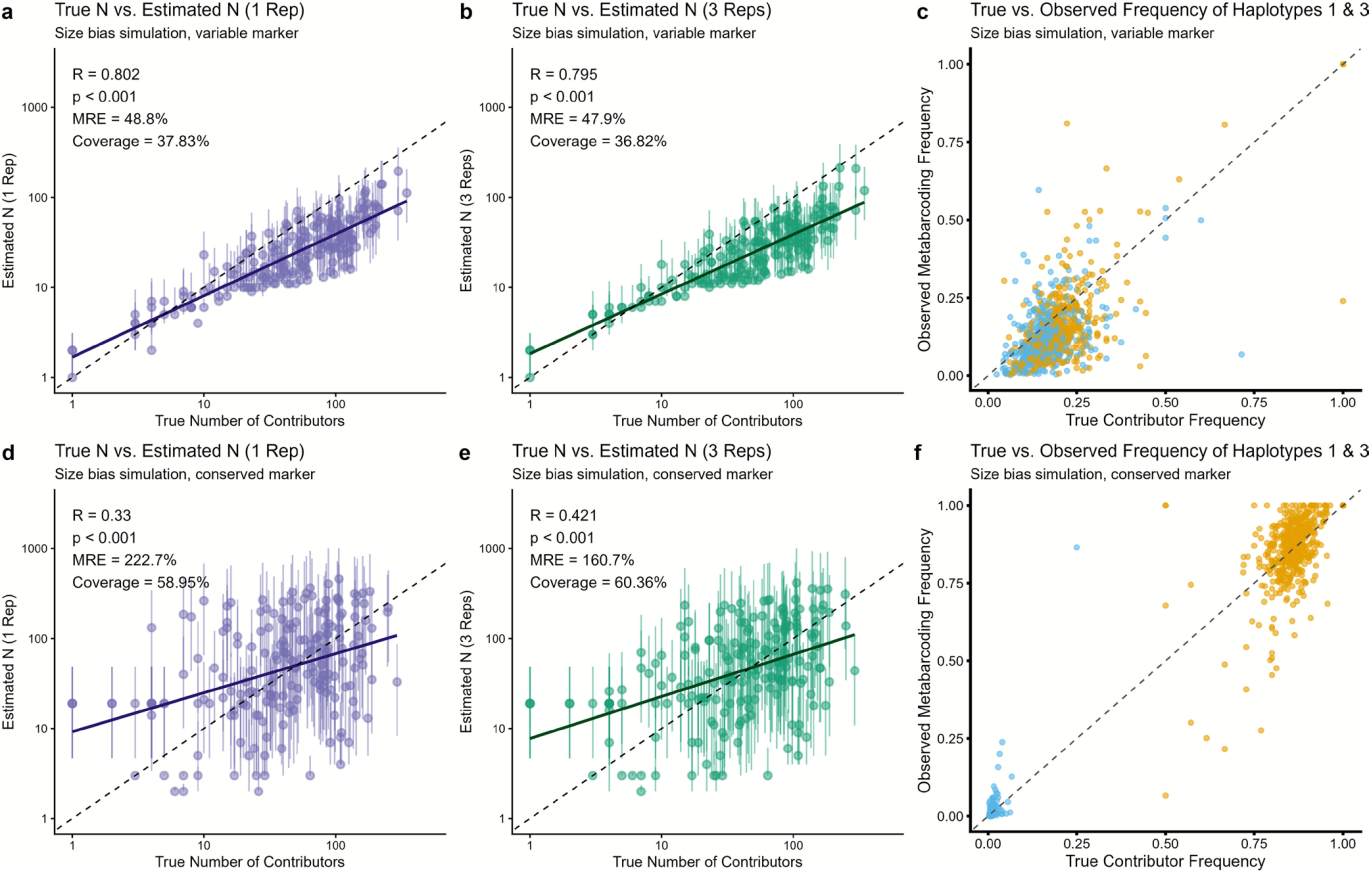
Precision of estimation methods applied to simulated data but with variable individual contribution (ξ > 1). Panels (a–c) show results from a simulation using a hyper‐variable marker with 17 alleles (same frequencies as Figure 5a,b) but with variable contribution from individuals including variable size (1–100) and distance from sample (1–100). (a) Estimation method applied to a single sample replicate with the hypervariable marker; (b) estimation method applied to three sample replicates with the hypervariable marker; (c) correlation between simulated haplotype frequencies and the observed haplotype frequencies for haplotypes 1 (yellow) and 3 (light blue), demonstrating that in these simulations, each contributor provided variable amounts of eDNA to the sample resulting in differences between true and observed frequencies. Panels (d–f) present a simulation using a conserved marker with 6 alleles (same frequencies as Figure 4d–f) but otherwise identical simulation to previous panels. (d) Estimation method applied to a single sample replicate with the conserved marker; (e) estimation method applied to three sample replicates with the conserved marker; (f) same as panel c but with conserved marker. Error bars in panels (a), (b), (d) and (e) are the estimate 95% CI (Equation 10). Solid lines in all panels represent the linear regression fits, *R* is Pearson's correlation, and dashed lines denote the 1:1 identity. Simulations included 5000 samples. While correlations are devised from all simulations, only a random subset of 200 are plotted here to avoid overplotting.

We then repeated the simulations using both sets of allele frequencies, this time introducing high variability in individual eDNA contribution and some observation bias to assess how the methods perform under more realistic sources of error (Figure 6). This results in substantial differences between contributor haplotype frequencies and observed eDNA haplotype frequencies (Figure 6c,f). We then repeated the estimator, using either one (Figure 6a,d) or three (Figure 6b,e) biological replicates (independent bottle grabs simulated from the same local eDNA pool).

As expected, the method underestimated the number of contributors under this scenario, especially in the samples with most contributors. This is due to errors in observed haplotype frequencies getting misinterpreted as a lower number of contributors. Nonetheless, estimates remained reasonable under the variable marker (Figure 6a–c). When analysing a single biological replicate (Figure 6a), correlation with the true number of contributors dropped to *R* = 0.802 (a drop of −0.048), MRE increased to 48.8% (a change of only +9.4%), but coverage dropped more significantly, with the correct answer recovered in only 37% of confidence intervals.

As discussed above, when observation noise is present, biological replicates can theoretically help remediate its effects, as the downstream noise (all except for eDNA ecology, Figure 2c–e) can be averaged over. Accordingly, when considering three biological replicates (Figure 6b), MRE and correlation coefficients only recovered slightly. This is because the majority of differences between contributor haplotype frequencies and observed frequencies in the simulations were due to eDNA ecology (variable contribution from each individual) and not by laboratory and observation processes shared by replicates.

When using a combination of noise and a conserved marker (Figure 6d–f), the estimations were substantially worse. The correlation between true and estimated numbers of contributors dropped to only *R* = 0.33, but remained significant (*p* < 0.001) and coverage also dropped significantly to 58%. Much like the conserved marker, adding biological replicates had little effect in improving estimates (Figure 5e). Finally, for both sets of haplotype frequencies, the number of contributors was slightly overestimated at low N, which is caused by singletons of the most frequent haplotype.

## 4 Discussion

This study introduces a new framework for estimating the number of individual contributors to environmental DNA (eDNA) samples using within‐sample haplotype frequencies and known or inferred population‐level haplotype distributions. By leveraging the natural statistical convergence between observed and population haplotype frequencies, we show that it is possible to derive meaningful abundance estimates from eDNA metabarcoding data without directly relying on the quantity of eDNA molecules. Simulations confirm that the method performs well under idealised conditions and remains informative even when incorporating biologically realistic sources of noise such as variation in contributor size, distance and shedding rates, provided the marker is variable enough. Together, these results suggest that haplotype frequency patterns offer a largely untapped source of quantitative information in eDNA data, bridging the gap between traditional presence/absence interpretation of metabarcoding and more species‐specific quantitative approaches.

A key methodological advance is the use of within‐sample haplotype frequencies to estimate contributor abundance. Earlier models rely exclusively on the identity (Andres et al. 2021) or number (Ai et al. 2025) of haplotypes while ignoring the observed frequencies. Thus, our approach fully leverages the information present in the data, leading to more accurate and nuanced estimates. Notably, we do not explicitly model haplotype presence or combinatorial arrangements; instead, these effects are implicitly captured within the frequency patterns. This requires the assumption that observed frequencies are centred on the true frequencies of contributors. However, our simulations, which incorporate substantial and realistic levels of noise, show that the method is robust even when this assumption is moderately violated. Nonetheless, it is possible that the level of noise observed in real samples goes beyond that, hindering applicability.

We also show that the method is broadly applicable across loci of varying haplotype diversity, but performs substantially better when applied to hypervariable markers (Figures 5 and 6). This pattern mirrors that of previously proposed methods based on haplotype counts or identity alone (Andres et al. 2021; Yoshitake et al. 2019). The reliance on marker variability presents a challenge for applying the method to many existing eDNA datasets, as commonly used markers (e.g., 12S and COI) are relatively conserved. This tradeoff reflects a tradeoff within eDNA metabarcoding: to detect a broad range of taxa, primers must target conserved regions, which limits taxonomic resolution and the richness of quantitative signals. However, as sequencing technologies advance and longer reads can be reliably obtained from eDNA, more variable sites will be recoverable, increasing the power of haplotype‐based inference.

In addition to estimating the number of contributors to individual eDNA samples, we also provide a framework for estimating population haplotype frequencies directly from eDNA data. While existing tissue‐derived reference datasets could be used, estimating *π* from eDNA offers several methodological advantages. First, it ensures internal consistency: the same eDNA data used to estimate contributors also defines the reference frequencies. This alignment naturally captures any dataset‐specific amplification or sequencing bias – biases that tissue‐derived haplotype frequencies may miss, especially when primer mismatches or multiple sequencing runs are involved. Second, eDNA often recovers haplotypes that are rare or absent from tissue datasets (e.g., Parsons et al. 2025). When such haplotypes appear in subsequent samples, their lack of reference frequency renders them unusable unless population frequencies are inferred from the eDNA data itself. While no single sample is expected to capture complete haplotype diversity, pooled frequencies across spatially and temporally replicated samples can serve as a biologically and methodologically coherent proxy. This approach mirrors traditional population surveys, where reliability emerges from replication, not from any single observation.

### 4.1 Practical Considerations and Method Limitations

In practical terms, we recommend applying this framework under the following conditions: first, population haplotype frequencies should be reasonably well approximated by multiple eDNA detections (> 20), ideally through replication across spatial or temporal scales. Second, sequencing depth and template DNA should be sufficient so that haplotype frequencies retain information about contributors rather than being dominated by noise. Third, markers with adequate intraspecific variability should be chosen, and caution is warranted with conserved loci such as 12S that provide limited resolution. These technical considerations operate alongside deeper biological assumptions about population structure and independence, which we address below.

Importantly, our method relies fundamentally on the assumption that eDNA contributors represent independent random draws from an unstructured population (panmixia). However, this assumption can be easily violated when natural populations have wide geographic ranges and exhibit population structure. For instance, in populations with pronounced geographic structure, local samples may reflect haplotype frequencies that deviate systematically from the overall population frequencies (e.g., Parsons et al. 2013, 2025), which would lead to biased contributor estimates if compared against global frequencies. Thus, local population frequencies are preferred if available. It is also common that invasive or expanding species will show spatial sorting in the edges of their distribution (Clarke et al. 2019; Comerford et al. 2023; Ochocki and Miller 2017), which will break the panmixia assumption. In addition, in species with strong social bonds or family groups, eDNA samples might capture genetic material from related individuals whose haplotypes are not independent. This is particularly problematic when analysing mitochondrial markers, which follow strict maternal inheritance patterns. In such cases, our model would be unable to distinguish between different scenarios with the same genetic composition. For example, a single adult female versus a mother–calf pair sharing identical mitochondrial haplotypes, or a school of fish fry who are all siblings. These biological realities highlight the importance of careful marker selection and thoughtful interpretation of results. Nonetheless, it is important to state that this reliance on panmixia is not unique to our framework, but reflects the simplifying baseline of many classical population genetic models like F‐statistics (Nei 1973) and Kingman coalescent model (Hudson 1990; Kingman 1982). Such assumptions rarely hold perfectly in nature, yet these models remain useful because they provide a tractable reference point: deviations introduce bias but do not eliminate all inferential value. Future extensions of the framework may likewise relax this assumption to accommodate structured populations.

As discussed in the Introduction, a common limitation of approaches for estimating the number of contributors, including those presented here and those developed by Andres et al. (2021); Andres, Lodge, and Andrés (2023); Andres, Lodge, Sethi, and Andrés (2023), is their reliance on population‐level haplotype frequencies. In this study, we suggest approximating these frequencies using the distribution of haplotypes across multiple eDNA samples, treating the pooled frequencies as a proxy for population‐level values. While we show this approach results in rapid approximation of true frequencies (Figure 4), this must be done carefully. First, one must be aware of the potential inclusion of false or artifactual haplotypes caused by PCR or sequencing errors. To mitigate this risk when applying the methods herein to real datasets, it is important to use filtering methods that ensure observed sequence variants indeed relate to haplotypes. Even with stringent bioinformatic pipelines, false alleles may persist in eDNA data (Tsuji et al. 2020). Fortunately, tools like lulu (Frøslev et al. 2017) can collapse erroneous variants by clustering sequences based on similarity and co‐occurrence, especially when calibrated with a mock community. More recently, dedicated R packages such as TombRaider (Jeunen et al. 2024) and gmmDenoise (Koseki et al. 2025) have introduced statistical filtering tailored to eDNA data, which also remove spurious ASVs. Together, these methods make it practical to remove artefacts and recover reliable haplotypes from environmental samples.

To date, most empirical work on eDNA population inference has been carried out in aquatic environments (Ai et al. 2025; Andres, Lodge, and Andrés 2023). Whether our proposed framework applies to terrestrial or aerial eDNA remains uncertain, as the ecology of DNA in these systems is far less well characterised. Airborne samples in particular often yield heterogeneous and relatively low DNA concentrations compared to water, conditions that may limit the recovery of haplotype frequency information across multiple contributors (Clare et al. 2022; Ip et al. 2025; Lynggaard et al. 2022). Under such circumstances, frequency‐based approaches like the one presented here may be less informative, and methods that rely on haplotype identity or counts (e.g., Andres et al. 2021; Andres, Lodge, and Andrés 2023; Andres, Lodge, Sethi, and Andrés 2023) are likely to be more appropriate. Clarifying the applicability of these alternative frameworks across different sample types will require targeted empirical studies.

Finally, when it comes to interpreting the results, it is crucial to emphasise that the derived contributor numbers should not be interpreted as exact counts of individual organisms in an area. Rather, they represent a conservative estimate of contributors whose DNA was captured and retained through the sampling and sequencing process. This value relates only indirectly, and likely through a complex, nonlinear function, to true organismal abundance. For example, if two lakes are sampled for eDNA and one lake yields an estimated 10 contributors and another 100, it may be reasonable to infer that the latter harbours more individuals overall, but not necessarily exactly 100 individuals or even 10 times as many as the first. The method is best interpreted as providing a conservative lower bound on contributor number, excluding key real‐world sources of variance such as differential shedding rates, environmental degradation and sequencing bias.

### 4.2 Future Steps and Validation

Here, we only applied the method to simulated eDNA samples, but further method validation with field and real eDNA samples is necessary and encouraged prior to broad method application, as the level of ‘noise’ added into real eDNA samples could be too high for this to be applicable in practice. The ideal dataset to validate the approach herein would include a population with known haplotype frequencies, a known or independently estimated number of contributing individuals, spatially distributed sampling and eDNA sequencing with a hypervariable marker. To our knowledge, no publicly available dataset meets all of these criteria, and generating such a dataset to apply the method as a proof of concept is beyond the scope of this study. Most existing eDNA metabarcoding efforts rely on relatively conserved mitochondrial loci – such as 12S, COI, or CytB – where the proposed method would work but with very low accuracy (Figures 4 and 5). Thus, while we chose to present and apply the method with simulated data to demonstrate the concept, future work should use empirical data to verify model effectiveness.

Future refinements or iterations of this approach should also explore ways to estimate ξ more reliably, so the underestimation bias under noisy scenarios can be corrected for, and the method made more reliable for large contributor numbers. In addition, future studies could also improve abundance estimation by integrating haplotype‐based inference with other independent data streams. One promising avenue is to combine this approach with total eDNA quantification (e.g., from qPCR or dPCR), which reflects bulk biomass but is subject to spatiotemporal biases (Ai et al. 2025). Because the two approaches capture distinct biological signals (number of individuals vs. biomass), they can provide complementary and independent estimates of abundance (see Figure S5). In principle, a sample with high eDNA quantity but low haplotype diversity suggests many molecules from few individuals, whereas high values for both suggest contributions from many individuals. Additionally, running the method across multiple unlinked loci could further improve robustness, especially in diploid or recombining organisms. This can be done by multiplying the likelihood distribution across loci estimates. While we did not explore such integrative frameworks here, they represent a clear next step towards more accurate and biologically meaningful estimation of population abundance from eDNA.

## Author Contributions

P.F.P.B.‐D. conceived the study. All authors contributed to the development of methods and statistical approaches. P.F.P.B.‐D. wrote the first draft of the manuscript, and all authors provided edits and approved the final version. E.A.A. and R.P.K. secured funding.

## Funding

This work was supported by the Office of Naval Research, N00014‐22‐1‐2719.

## Disclosure

This research contributes benefits by making all code publicly available through the databases described above.

## Conflicts of Interest

The authors declare no conflicts of interest.

## Supporting information


**Data S1:** men70104‐sup‐0001‐DataS1.zip.
**Figure S1:** Mechanisms driving the convergence of observed eDNA haplotype frequencies.
**Figure S2:** Detailed propagation of variance in haplotype frequencies observed within an environmental DNA sample.
**Table S1:** Main text and figure S2 mathematical notations (in alphabetical order) and their respective units and categories.
**Figure S3:** The effect of various haplotype frequencies (*π*) on effectiveness of estimations.
**Figure S4:** Comparison Between Normal Approximation MLE and Method of Moments (MoM).
**Figure S5:** Simulation on figure illustrating how haplotype number and eDNA concentration can provide conflicting but complementary information.

## Data Availability

All code necessary to replicate the simulations and a function to apply the likelihood method is made available in a qmd file in https://github.com/pedrobdfp/eDNA_haplo_frequencies, and as a supplement.
