# Supplementary material for: Estimating Organism Abundance Using Within‐Sample Haplotype Frequencies of eDNA Data
Source: Mol Ecol Resour. 2026 Feb 13;26(2):e70104. doi: 10.1111/1755-0998.70104 (PMC12902907; doi:10.1111/1755-0998.70104)
Supplement: Supplementary file 1 — Data S1: men70104‐sup‐0001‐DataS1.zip. Figure S1: Mechanisms driving the convergence of observed eDNA haplotype frequencies. Figure S2: Detailed propagation of variance in haplotype frequencies observed within an environmental DNA sample. Table S1: Main text and figure S2 mathematical notations (in alphabetical order) and their respective units and categories. Figure S3: The effect of various haplotype frequencies (π) on effectiveness of estimations. Figure S4: Comparison Between Normal Approximation MLE and Method of Moments (MoM). Figure S5: Simulation on figure illustrating how haplotype number and eDNA concentration can provide conflicting but complementary information. [file MEN-26-e70104-s001.zip › men70104-sup-0001-FigureS1-S5-TableS1-S1.pdf]

Supplementary Information for

Estimating Organism Abundance Using Within-Sample Haplotype Frequencies of eDNA Data

Pedro FP Brandão-Dias[1*]; Gledis Guri[1]; Megan Shaffer[1]; Elizabeth Andruszkiewicz Allan[1]; Ryan P Kelly[1]

1: School of Marine and Environmental Affairs, University of Washington, Seattle, Washington, United States of America

*Corresponding Author: pedrobdfp@gmail.com

Table of Contents

Figure S1 2

Figure S2 3

Table S1 4

Figure S3 5

Method of Moments 6

Figure S4 7

Figure S5 8

References 9

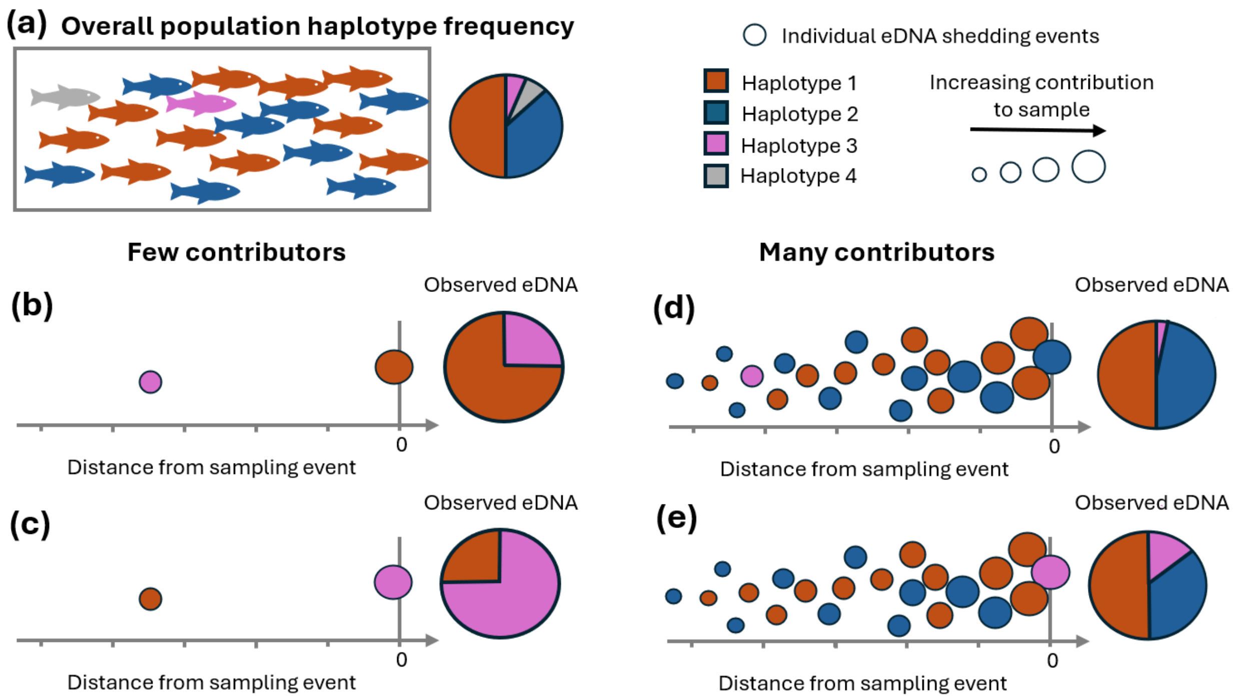

Figure S1: Mechanisms driving the convergence of observed eDNA haplotype frequencies toward population frequencies. (a) Population-wide haplotype frequencies: The pie chart on the right depicts the population haplotype frequencies, corresponding to the distribution of haplotypes (Haplotypes 1–4) represented by colored fish. (b-e) Individual eDNA shedding events: Circles represent individual eDNA shedding events, with circle size indicating their relative contribution to the sample, which is given by their spatiotemporal distance from the sampling location (distance = 0). (b, c) Small number of contributors: With less contributors, the observed haplotype frequencies are quite different from the population frequencies. In (b), Haplotype 1 (orange) dominates due to a nearby shedding event, causing observed haplotype frequencies to deviate significantly from population frequencies. Similarly, in (c), Haplotype 3 (pink) is overrepresented because of proximity to the sampling location. (d, e) Large number of contributors: With more contributors, eDNA from a greater diversity of individuals is captured, and observed haplotype frequencies increasingly align with population frequencies. The influence of a single contributor with Haplotype 3 (pink) becomes less pronounced, whether the individual is far from (d) or close to (e) the sampling location. Therefore, when fewer contributors are present (b, c), a single shedding event's position can have a disproportionate effect. In contrast, with many contributors (d, e), the aggregated contributions make the relative position of each individual event less impactful.

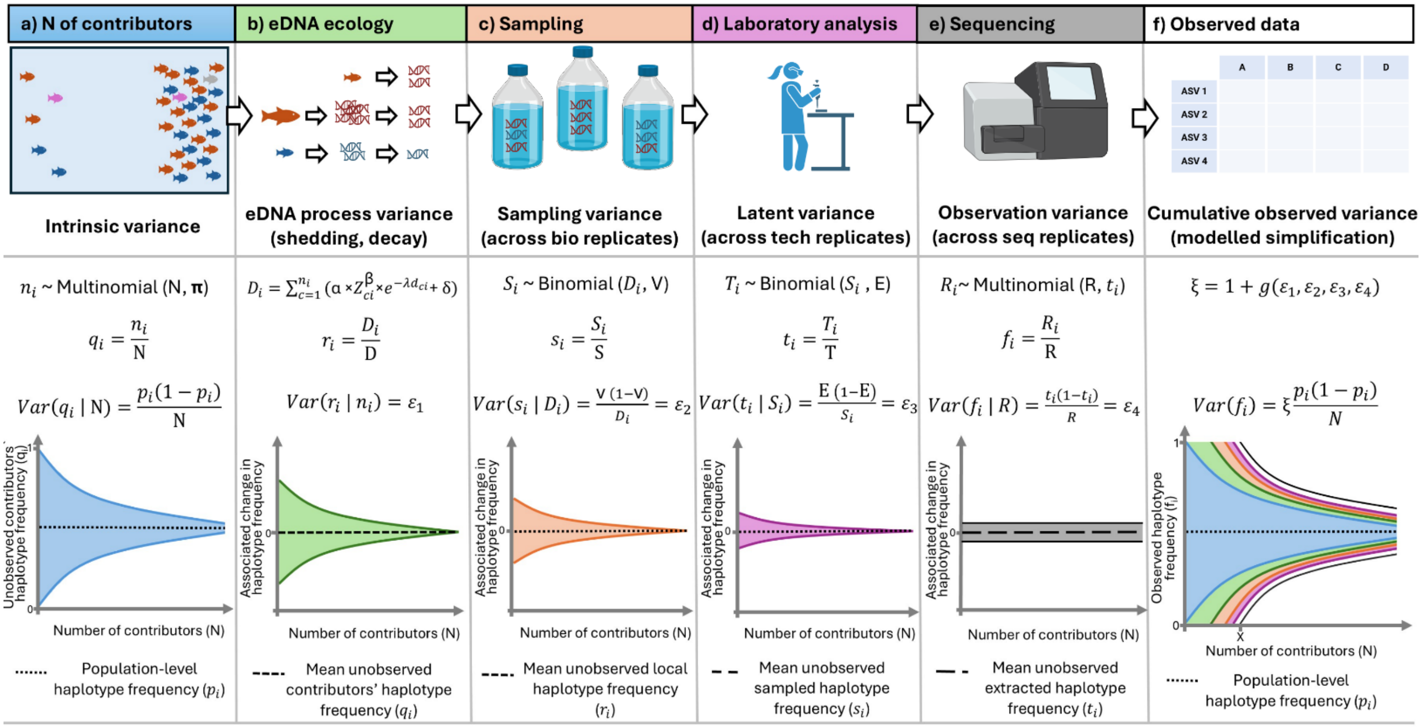

Figure S2: Detailed propagation of variance in haplotype frequencies observed within an environmental DNA sample. Note: All notations explained in Table S1 below. The sample index j (used in the main text to denote independent samples) is omitted here for simplicity, as the figure illustrates variance propagation for a single representative sample. Panel (a) illustrates the inherent process of eDNA production and distribution in the natural environment, where the variability in haplotype frequencies is intrinsically higher in regions with fewer contributors (*N*). This baseline variance allows one to infer the original number of contributors by examining haplotype frequency variability across samples. In panel (b) we observe the eDNA process variance, where different individuals (and thus biomasses) result in different shedding rates, or distance (in space or time) between an individual contributor and the sample results in reduced eDNA concentrations from either decay or transport. Thus, individual contributors provide a variable amount of eDNA to the local DNA pool D, affecting local haplotype frequencies (r_i). By "local pool" we mean the entire eDNA pool in a given area that could be sampled. Panel (c), D_i are sampled, yielding intermediate, unobserved sampled frequencies for each haplotype (s_i). Panel (d) depicts the laboratory processes—such as DNA extraction, pipetting—that further add noise, resulting in extracted haplotype frequencies (t_i). In panel (e), the sequencing step is represented as a multinomial sampling process, where the variance of the observed haplotype frequencies (f_i) is a function of the intermediate frequencies (t_i) and is inversely proportional to the sequencing read depth (R); deeper sequencing results in lower variance at this step. Finally, panel (f) shows that the final observed haplotype frequencies incorporate the cumulative variance from all stages—from natural eDNA distribution through sampling, laboratory processing, and sequencing. Point X on the x-axis shows the N that is indistinguishable from 1 due to added noise. Created with BioRender.com

Table S1: Main text and figure S2 mathematical notations (in alphabetical order) and their respective units and categories

Symbol	Description	Category	Units
c	Index for contributor	**Index**	–
i	Index for haplotype	**Index**	–
j	Index for sample	**Index**	–
$d_{c,j}$	Distance between contributor c and sample j (in space or time)	**Model parameter**	arbitrary
$D_{i,j}$	Number of DNA molecules of haplotype i in the local eDNA pool for sample j	**Process variable**	copies
E	Extraction efficiency (proportion of molecules extracted)	**Model parameter**	–
$f_{i,j}$	Proportion of reads observed for haplotype i in sample j	**Observed variable**	–
N_j	Total number of contributors to eDNA sample j	**Variable of interest**	individuals
$n_{i,j}$	Number of individual contributors with haplotype i contributing to sample j	**Process variable (stochastic)**	individuals
p_i	"True" population frequency of haplotype i	**Latent variable**	–
$q_{i,j}$	Unobserved frequency of haplotype i among contributors to sample j	**Latent variable**	–
$r_{i,j}$	Proportion of DNA molecules of haplotype i within the local eDNA pool for sample j	**Derived variable**	–
$R_{i,j}$	Number of sequenced DNA molecules (reads) of haplotype i in eDNA sample j	**Observed variable**	reads
R_j	Total number of sequenced DNA molecules (reads) in sample j	**Observed variable**	reads
$s_{i,j}$	Proportion of DNA molecules of haplotype i within sample j	**Derived variable**	–
$S_{i,j}$	Number of eDNA molecules of haplotype i in sample j	**Process variable**	copies
$t_{i,j}$	Proportion of extracted DNA molecules of haplotype i in sample j	**Derived variable**	–
T_j	Number of eDNA molecules extracted from sample j	**Process variable**	copies
V_j	Proportion of the local eDNA pool captured in sample j	**Model parameter**	–
Z_c	Size (biomass) of contributor c	**Model parameter**	arbitrary
α	eDNA shedding coefficient	**Model parameter**	copies per size unit
β	Allometric scaling parameter	**Model parameter**	–
δ	Random variation in eDNA shedding among contributors	**Stochastic term**	–
$\varepsilon_1 \dots \varepsilon_4$	Variance terms added at each process (ecology, sampling, extraction, sequencing)	**Stochastic term**	–
λ	eDNA decay rate	**Model parameter**	rate per distance unit
ξ	Multiplicative variance inflation term	**Stochastic term**	–
$\pi = \{p_1, \dots, p_I\}$	Vector of "true" population haplotype frequencies	**Latent variable**	–

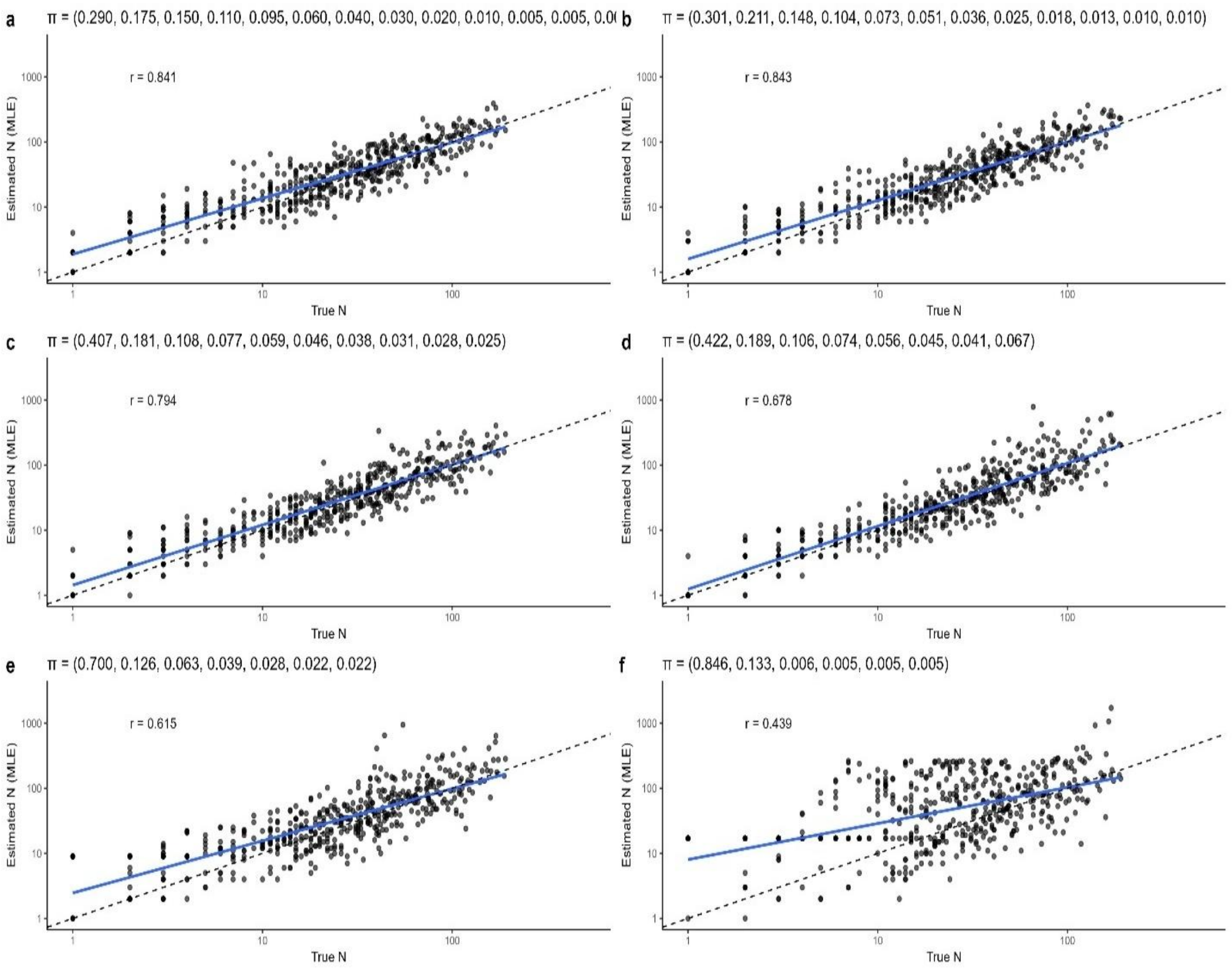

Figure S3: The effect of various haplotype frequencies (π) on effectiveness of estimations. π above each pair of panels show the haplotype frequencies in the simulations. R is Pearson's correlation, dashed line is the 1:1 line. Simulations performed with no error to determine maximum effectiveness of the method

Method of Moments Approach

An alternative to the normal approximation maximum likelihood approach presented in the main text is the method-of-moments approach. It is mathematically equivalent, as it stems from the same principle and the final derived equation is very similar. Nonetheless, the method does not provide a likelihood distribution, making it challenging to combine multiple loci. Additionally, preliminary tests showed that the Normal MLE approach performed better under most scenarios (Figure S3). Hence, it was presented in the main text in favor of this method. Nonetheless, we chose to present the method of moments solution here as well, as it may be useful for integration into other non-likelihood frameworks.

Under the multinomial model (Eq. 1-4 in the main text), the expected sum of squared deviations between the sampled frequencies f and the population frequencies π, that is, the difference between observed and population haplotype frequencies, is given by equation 7:

$$E\left[\sum_{i=1}^{K}(f_i - p_i)^2\right] = \frac{1 - \sum_{i=1}^{K} {p_i}^2}{N} \qquad (S1)$$

In essence, it states that as N increases, the observed frequencies f become closer to π, and the total deviation shrinks proportionally to 1/N. With known population frequencies, the square deviations of frequencies can be derived from samples:

$$S_{OBS} = \sum_{i=1}^{K}(f_i - p_i)^2 \qquad (S2)$$

Then, under the method of moments (Luikart et al., 1999; Osękowski, 2017; Waples, 1989), we equate the observed value S_{OBS} (Eq. S2) to its theoretical expectation that is a known function of the unknown parameter (Eq. S1) Therefore:

$$\sum_{i=1}^{K}(f_i - p_i)^2 \ = \frac{1 - \sum_{i=1}^{K} {p_i}^2}{N} \qquad (S3)$$

Solving for N, we obtain

$$N_{MoM} \ = \frac{1 - \sum_{i=1}^{K} {p_i}^2}{\sum_{i=1}^{K}(f_i - p_i)^2} \qquad (S4)$$

Solving this equation with the observed frequencies of all haplotypes in the sample will provide a point estimate for the expected number of contributors to the eDNA sample, given the observed frequency variance. To provide confidence intervals, we can approximate the uncertainty in N via the delta method (Dorfman, 1938). In our implementation, the standard error (SE) of N is approximated as

$$SE(N_{ij}) \approx \sqrt{\frac{2N^2}{n}} \qquad (S5)$$

Where n is the number of haplotypes with non-zero observed frequency. With this standard error, we construct a 100(1 – α) % confidence interval for N as

$$N_{ij} \pm z_{1-\alpha/2} \times SE(N_{ij}) \qquad (S6)$$

Where z is the z score from the normal distribution.

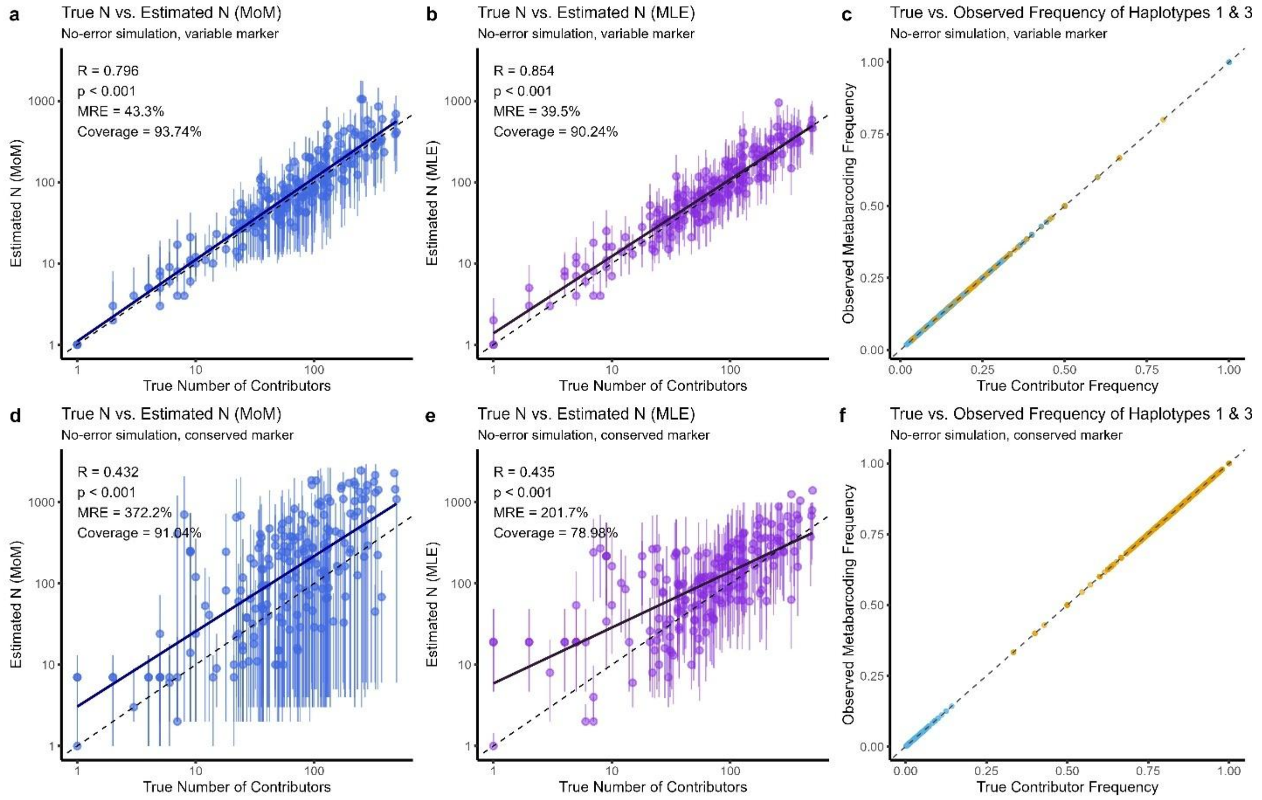

Figure S4: Comparison Between Normal Approximation MLE and Method of Moments (MoM). Panels (a–c) show results from a simulation using a hyper-variable marker. Panels (d-f) present a simulation using a conserved marker. In panels (a, d) estimates were derived using the Method of Moments (MoM, Equation S4); in panels (b, e) estimates were derived using the Normal-Approximation Maximum Likelihood method (main text); panels (c, f) show the correlation between simulated contributors haplotype frequencies and the observed haplotype frequencies in the corresponding simulated metabarcoding observations, demonstrating that in these simulations, little error is added. Solid lines in all panels represent the linear regression fits, R is Pearson's correlation, and dashed lines denote the 1:1 identity. Simulations included 5000 samples. While correlations are devised from all simulations, only a random subset of 200 are plotted here to avoid overplotting.

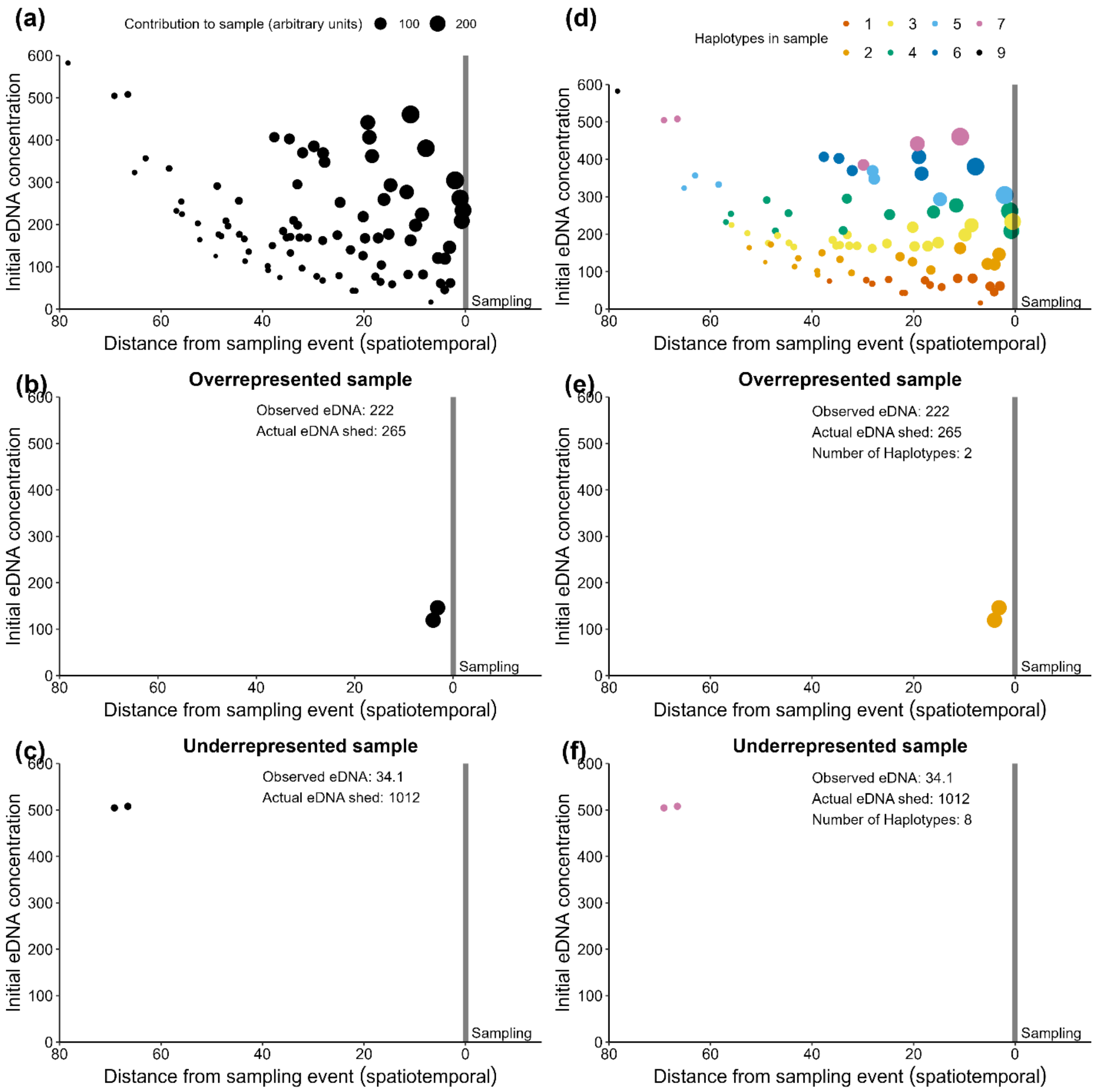

Figure S5: Simulation figure illustrating how haplotype number and eDNA concentration can provide conflicting but complementary information. In panel (a), each black dot represents an eDNA shedding event, varying in initial concentration and occurring at different distances—either spatially or temporally—from the sampling point. Consequently, when a sample is taken, these events contribute unevenly to the eDNA sample, leading to biased estimates that tend to favor the most recent or nearby shedding events. For instance, in panel (b), shedding events occur close to the sampling point, resulting in an overestimation of local target abundance if only DNA concentration is considered. Conversely, in panel (c), the shedding event occurs farther from the sampling point, leading to an underestimation of abundance if only concentration is considered. Therefore, if this bias can be corrected, then theoretically we would improve abundance estimates from eDNA data. As the number of individuals contributing to a sample increases, so

does the haplotype diversity, as shown in panel (d). Therefore, in a scenario like panel (e), where observed eDNA concentration is high but haplotype diversity is low, we may infer that the sample is overrepresented due to shedding events occurring close to the sampling point. Conversely, in panel (f), where eDNA concentration is low but haplotype diversity is high, it indicates that the sample is underrepresented due to shedding events occurring farther from the sampling point.

REFERENCES

Luikart, G., Cornuet, J.-M., & Allendorf, F. W. (1999). Temporal Changes in Allele Frequencies Provide Estimates of Population Bottleneck Size. *Conservation Biology*, *13*(3), 523–530. https://doi.org/10.1046/j.1523-1739.1999.98133.x

Osękowski, A. (2017). Chapter 1—Method of Moments and Sharp Inequalities for Martingales. In I. Pinelis (Ed.), *Inequalities and Extremal Problems in Probability and Statistics* (pp. 1–27). Academic Press. https://doi.org/10.1016/B978-0-12-809818-9.00001-X

Waples, R. S. (1989). A generalized approach for estimating effective population size from temporal changes in allele frequency. *Genetics*, *121*(2), 379–391. https://doi.org/10.1093/genetics/121.2.379
